# Aspiration of a Large Acromioclavicular Joint Cyst Complicated by Recurrence and Enlargement: A Case Report

**DOI:** 10.7759/cureus.34754

**Published:** 2023-02-07

**Authors:** Sherlyn Yen Yu Tham, Poh Hwee Julia Ng, Sean Kean Ann Phua, Sean Wei Loong Ho

**Affiliations:** 1 Department of Orthopaedic Surgery, Tan Tock Seng Hospital, Novena, SGP

**Keywords:** needle aspiration, rotator cuff tear, acromioclavicular joint arthropathy, geyser sign, acromioclavicular joint cyst

## Abstract

This case report describes a patient with an enlarging and painless lump over the right acromioclavicular joint (ACJ). MRI showed a synovial cyst superior to the ACJ with a concomitant full-thickness tear of the supraspinatus. The patient underwent needle aspiration of the lump, which yielded 100ml of gelatinous fluid with no microbe growth. Despite repeated aspirations, the ACJ cyst continually enlarged beyond its initial size. This case report describes an ACJ cyst that enlarged in size after needle aspiration. The authors suggest surgical alternatives if cyst recurrence is observed after the initial attempt of aspiration.

## Introduction

Acromioclavicular joint (ACJ) cyst is a rare condition that may occur in the elderly [1,2]. These cysts can result from localized ACJ pathology or degenerative shoulder joint pathologies such as rotator cuff tears [3]. In localized ACJ pathology, degeneration of the ACJ results in fluid accumulation superficial to the ACJ. Conversely, in degenerative shoulder joint conditions, the cyst occurs due to communication between the glenohumeral joint and ACJ. Synovial fluid flows out of the glenohumeral joint and into the ACJ. The cyst is created due to the presence of a “one-way valve”, which does not allow the fluid to return to the joint [4].

While this cyst is benign, it can cause significant distress to the patient. In addition to cosmetic concerns, these cysts can also interfere with daily activities and dressing when large. ACJ cysts can be treated conservatively or surgically [2]. There are a variety of proposed surgical treatments, such as cyst and distal clavicle excision [2,4-7], rotator cuff repair [8], and arthroplasty [3]. Conservative management includes needle aspiration [1,3,5,9], corticosteroid injections [6,10,11], or “watchful waiting” [12-14]. There is, to date, no established consensus on the ideal treatment of the ACJ cyst, although limited data suggest a lower recurrence rate with a surgical approach [2,5,6,15].

We present a case of a 71-year-old male with a right ACJ cyst who underwent needle aspiration of the cyst complicated by recurrence and subsequent enlargement.

## Case presentation

**Figure 1:**
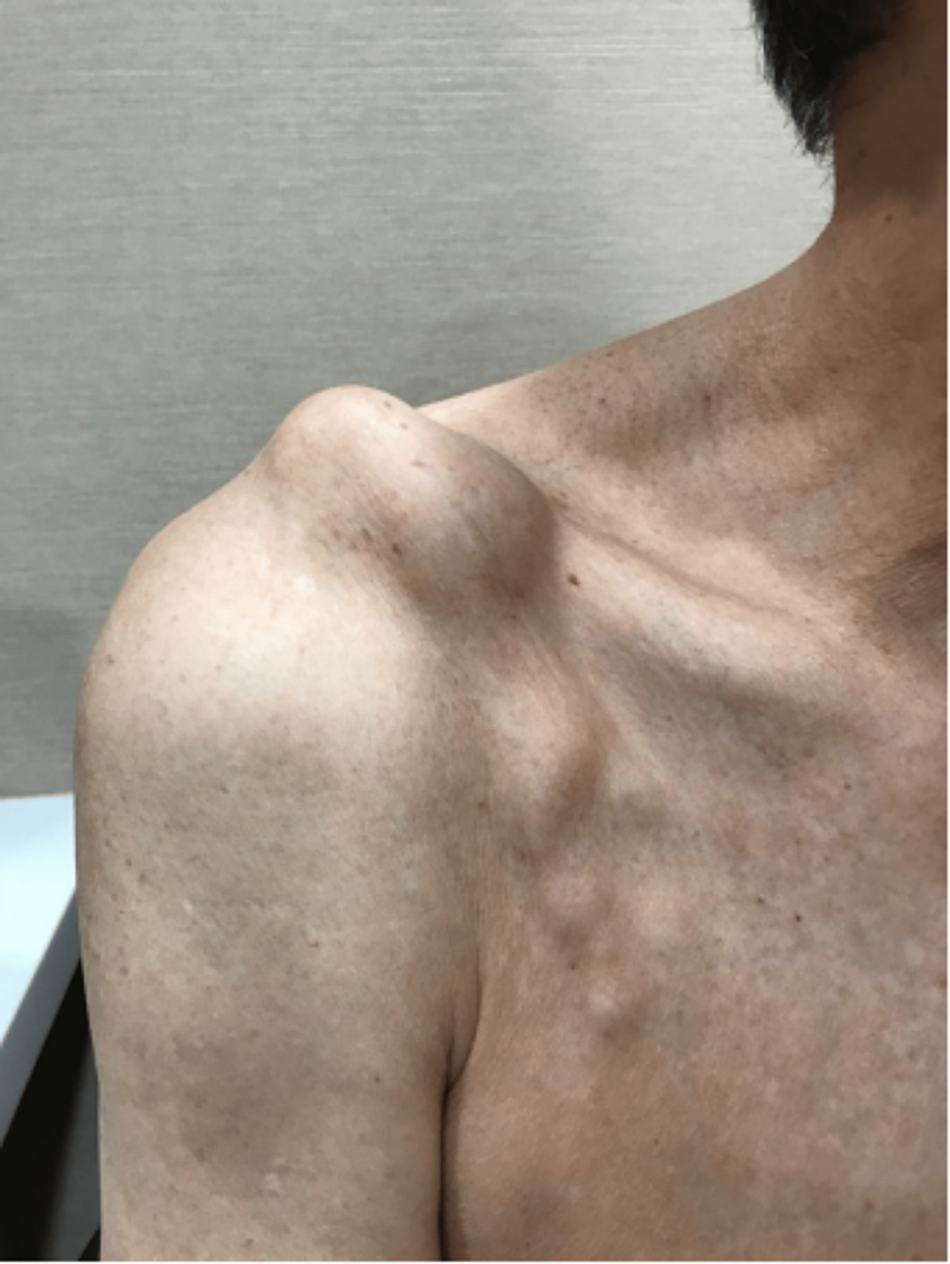
Clinical photograph of the right shoulder (anterior view) demonstrating a large cystic swelling anterosuperior to the ACJ.

**Figure 2:**
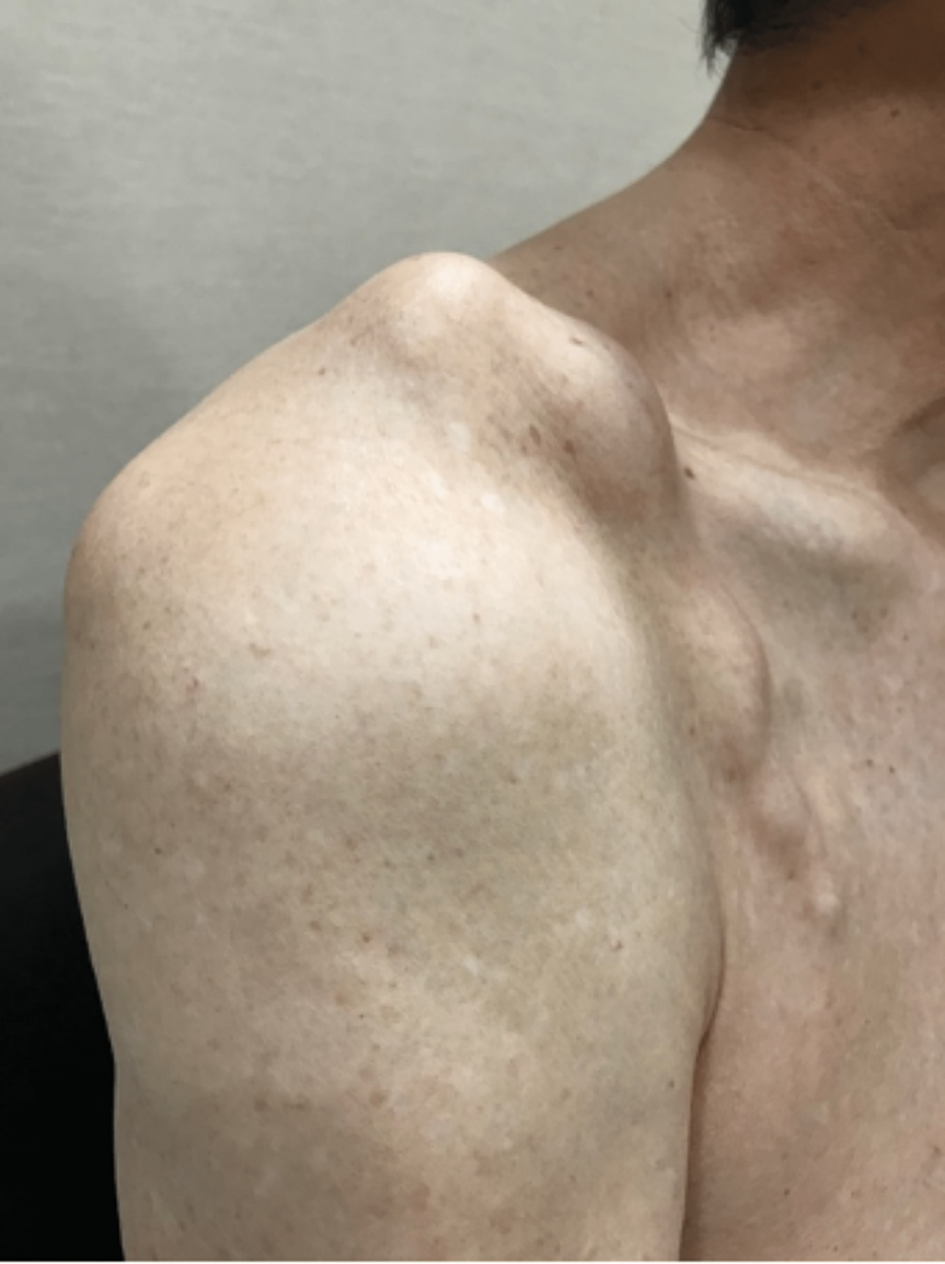
Clinical photograph of the lateral view of the right shoulder.

A 71-year-old male with no significant past medical history presented with a right shoulder lump located superior and anterior to the acromioclavicular joint, as shown in Figures 1, 2. This lump was enlarging in size over the past year. There was no pain in the shoulder. The patient had minimal limitations in daily living activities but complained of difficulty wearing his clothing. He was also concerned about cosmesis. The lump over the ACJ was non-tender and fluctuant. There was a reduced range of motion of the ipsilateral shoulder, with 0-140 degrees of abduction, 50 degrees of external rotation, and internal rotation up to the second lumbar vertebrae level.

**Figure 3:**
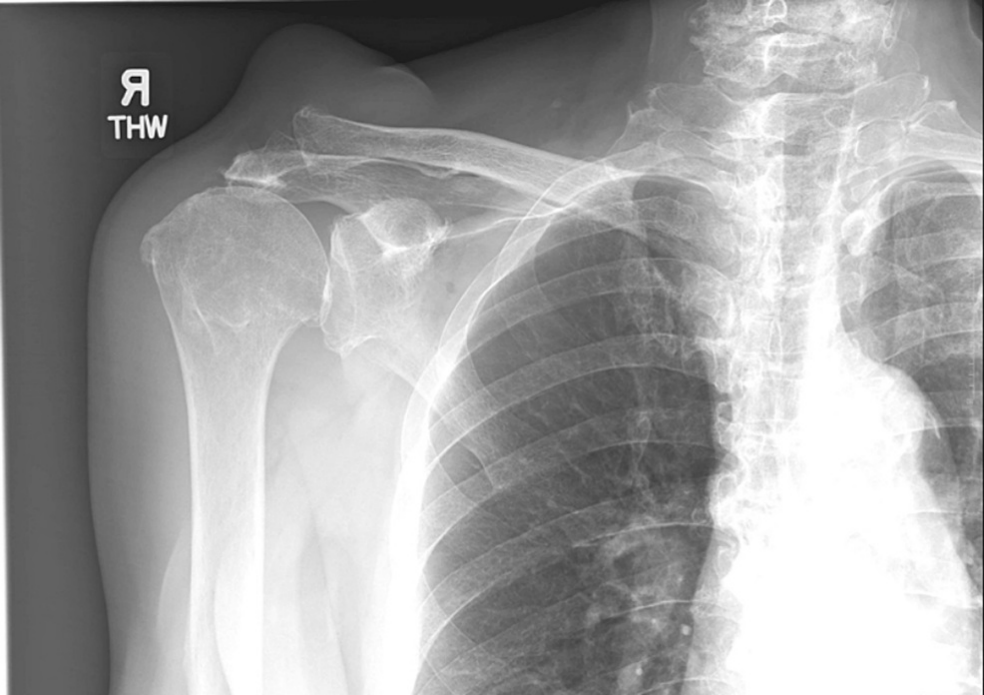
Plain radiograph of the right shoulder (AP view) showing a high riding humerus with decreased acromiohumeral distance and soft tissue swelling over ACJ. ACJ: acromioclavicular joint

**Figure 4:**
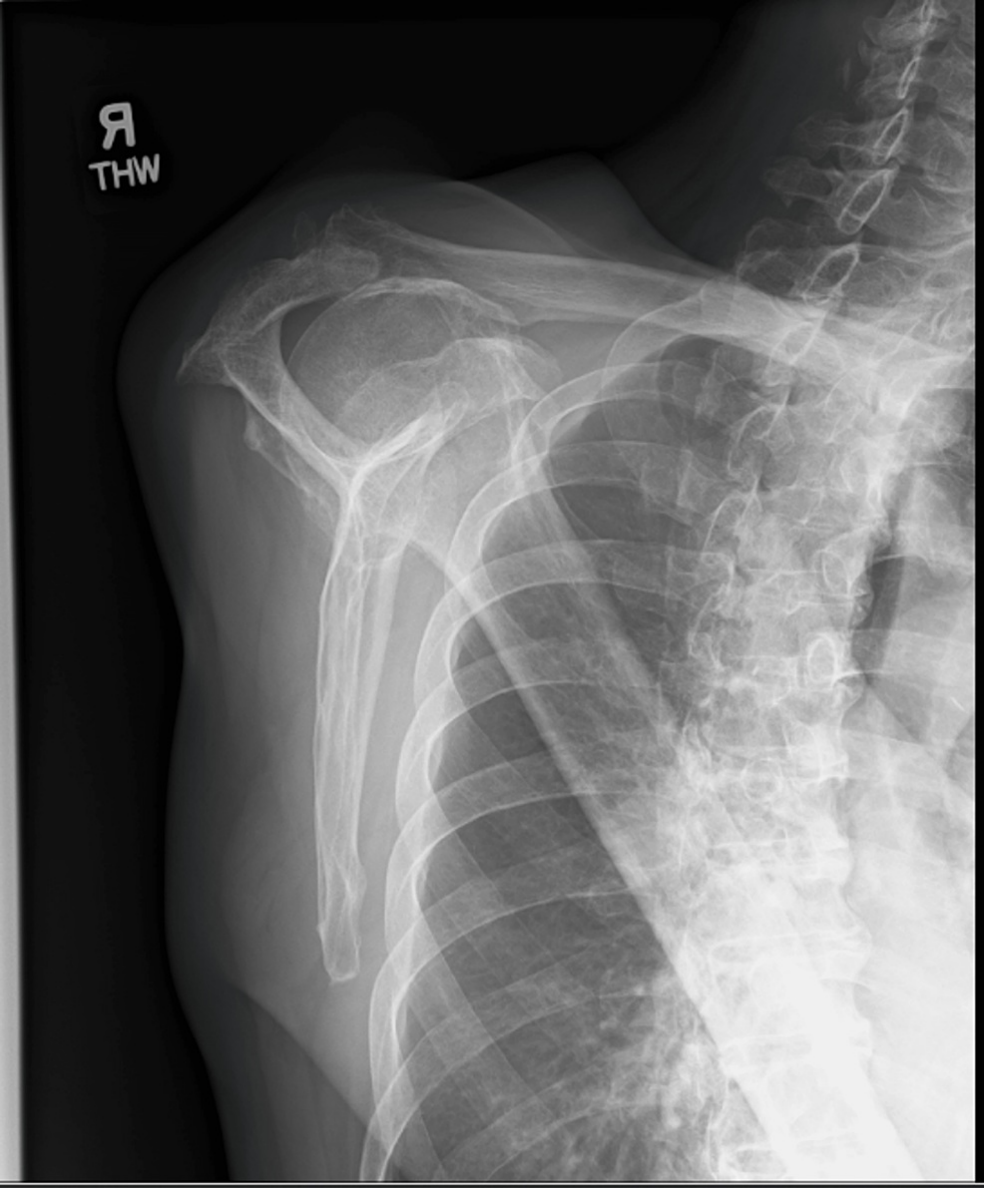
Plain radiograph of the right shoulder (Y-scapula view).

A plain radiograph revealed rotator cuff arthropathy with superior migration of the right humeral head. A soft tissue swelling superior to the right acromioclavicular joint was noted (Figures 3, 4).

**Figure 5:**
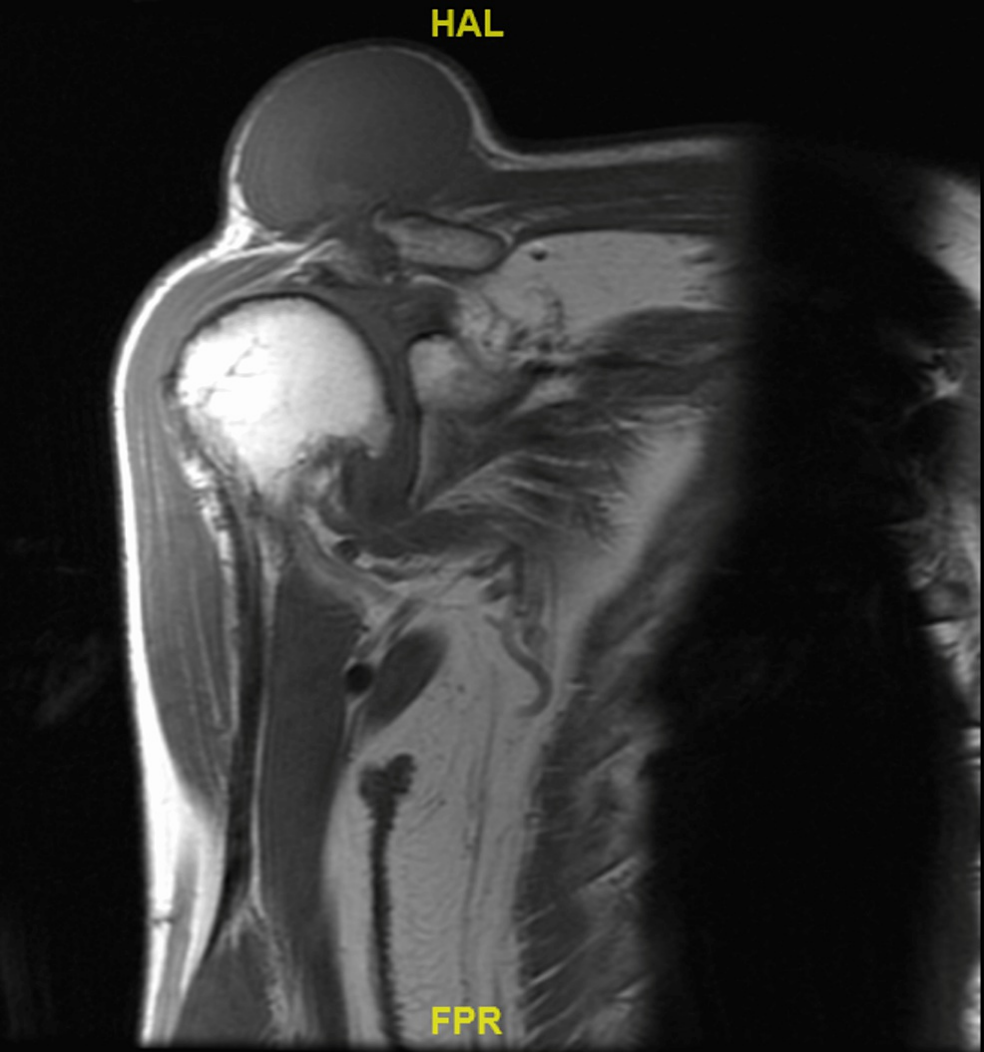
Coronal MRI of the right shoulder (pre-contrast T1-weighted sequence) demonstrating a cystic structure overlying the acromioclavicular joint. The high-riding humerus with reduced acromiohumeral distance is again shown.

**Figure 6:**
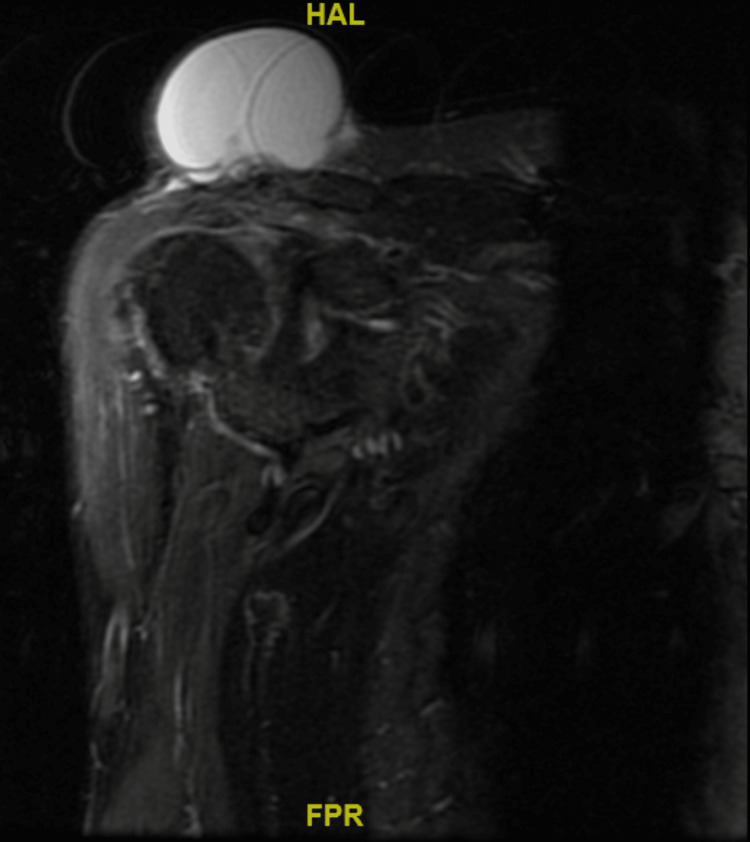
Coronal MRI of the right shoulder inversion recovery sequence.

**Figure 7:**
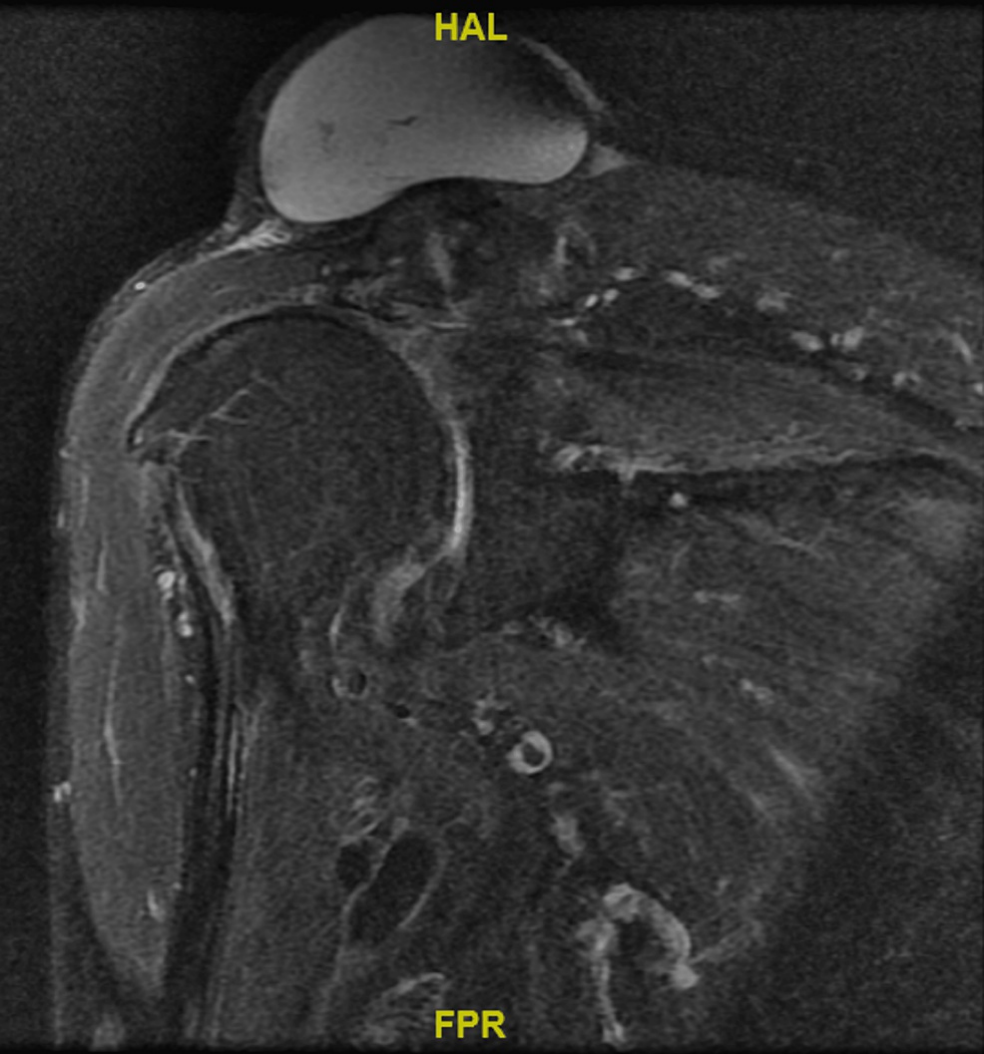
Coronal MRI of the right shoulder proton density fat-saturated sequence.

A MRI scan was performed. A non-contrast scan was performed as the lesion was clinically assessed to be an ACJ cyst, aiming to determine rotator cuff and joint-level pathology. MRI showed a complete full-thickness tear of the supraspinatus with a high-riding humeral head. Tendinopathy and atrophic changes were noted in the rest of the rotator cuff muscles. There was also severe acromioclavicular joint arthrosis with a synovial cyst measuring 7.6x6.2x4.7 cm (height) superior to the acromioclavicular joint (Figures 5-7). The presence of small cystic swellings over the anterior shoulder was also seen, and it was noted to be a continuation of the ACJ cyst.

**Figure 8:**
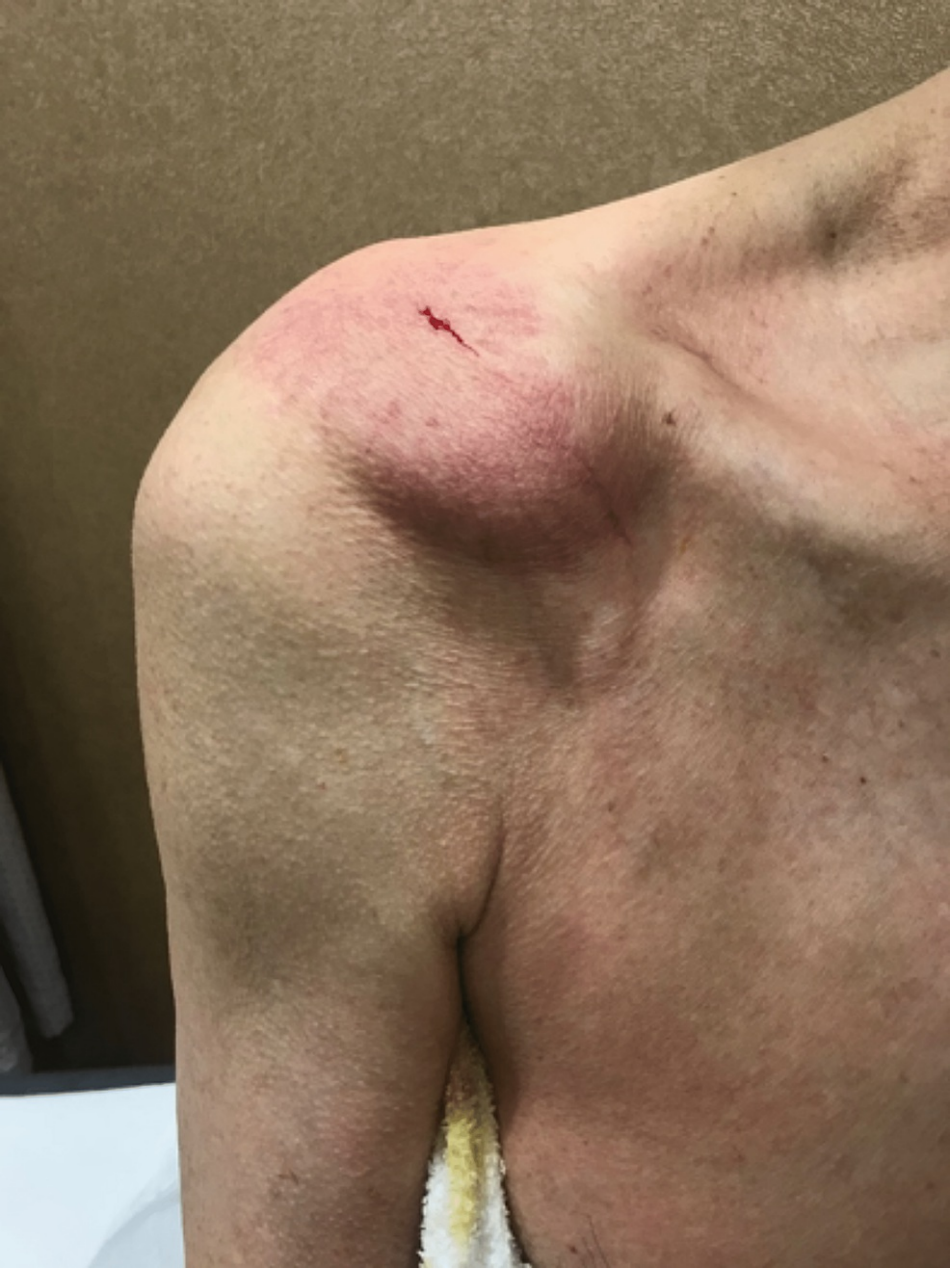
Clinical photograph post aspiration of right ACJ cyst (anterior view) showing a reduction in the size of ACJ cyst post aspiration. ACJ: acromioclavicular joint

**Figure 9:**
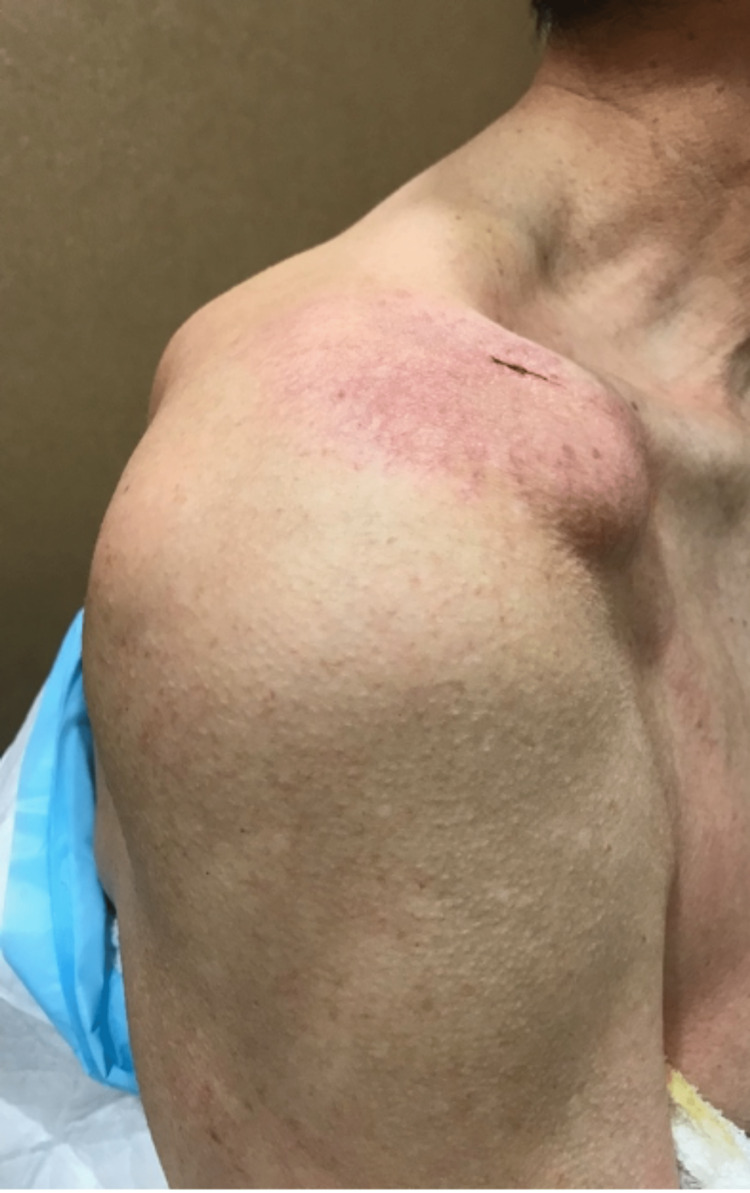
Clinical photograph post aspiration of right ACJ cyst (lateral view). ACJ: acromioclavicular joint

**Figure 10:**
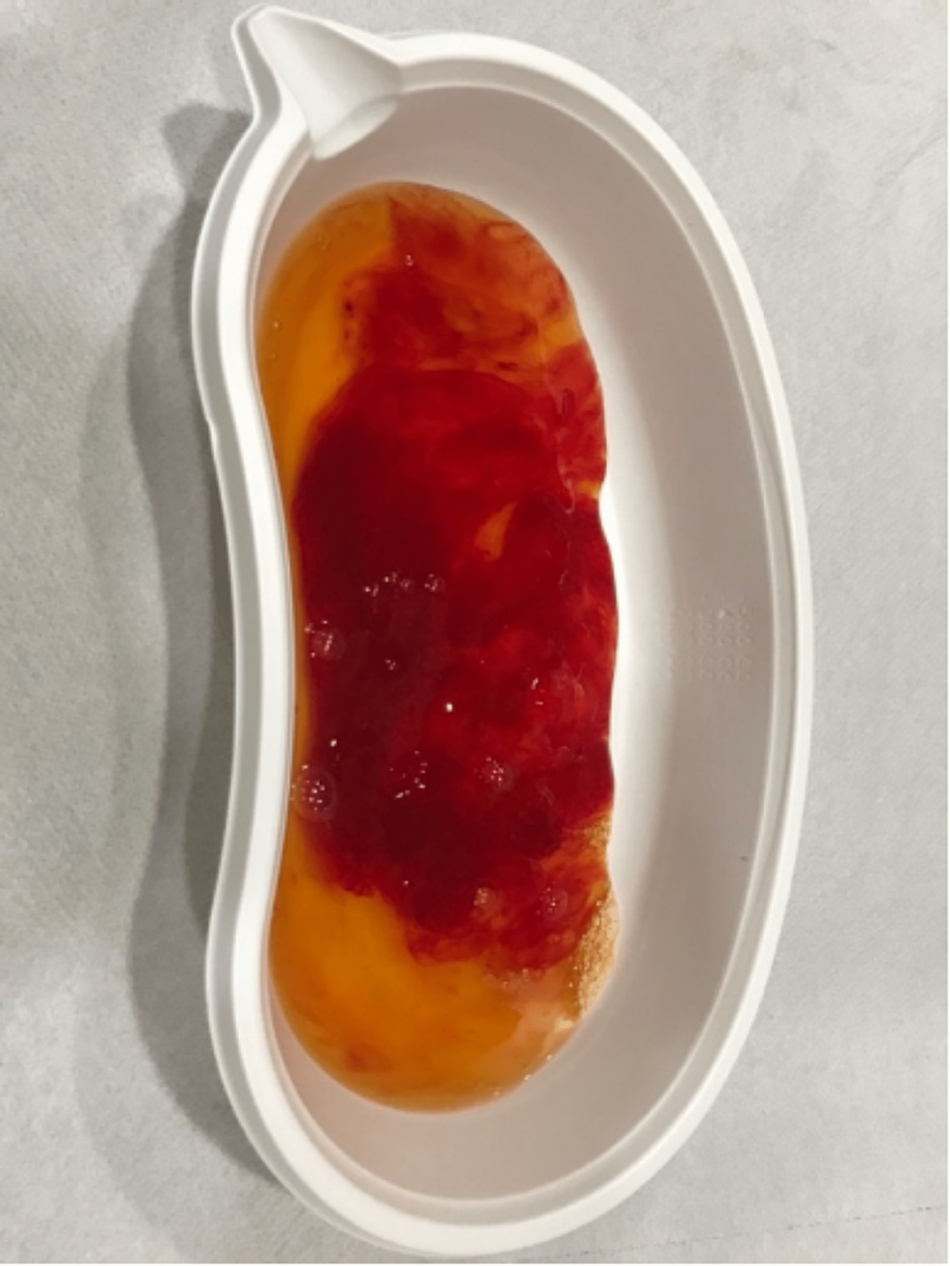
Clinical photograph showing hemogelatinous cyst aspirate contents.

Management options were discussed with the patient. The surgical options offered included an arthroscopic rotator cuff repair with distal clavicle excision or reverse shoulder arthroplasty. However, the patient was keen on non-surgical treatment and opted for needle aspiration of the cyst. Aspiration of the cyst was performed in the outpatient setting. Clinical photographs of the ACJ cyst post-aspiration are shown in Figures 8, 9. 100mL of gelatinous fluid was aspirated (Figure 10). Post-procedure, the patient had a pressure dressing applied for 48 hours and was allowed to range the shoulder as tolerated. The gram stain and cultures of the aspirated fluid did not show any microbial growth. Cell counts and cytology were not sent as the lump appeared clinically benign.

**Figure 11:**
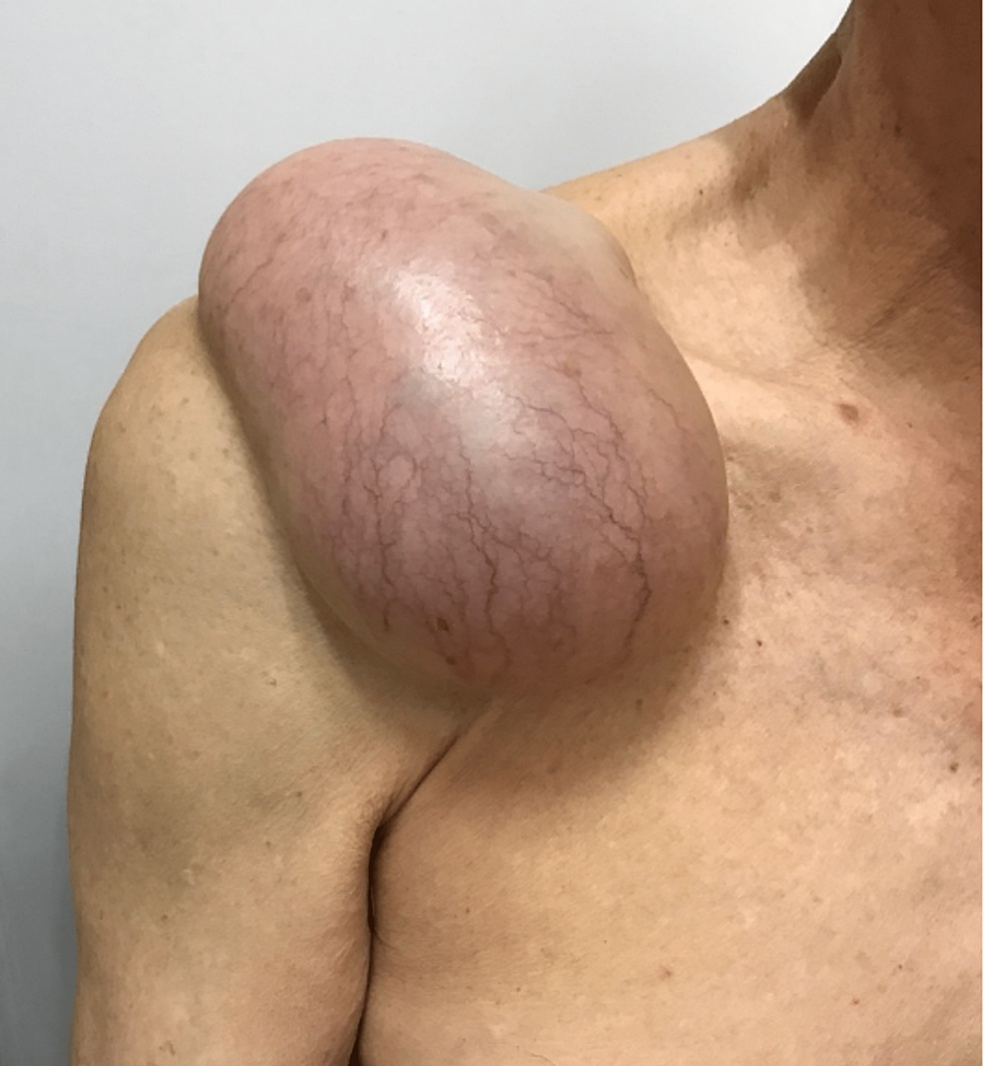
Clinical photograph showing recurrence of right shoulder ACJ cyst with progressive enlargement in size post needle aspiration. ACJ: acromioclavicular joint

**Figure 12:**
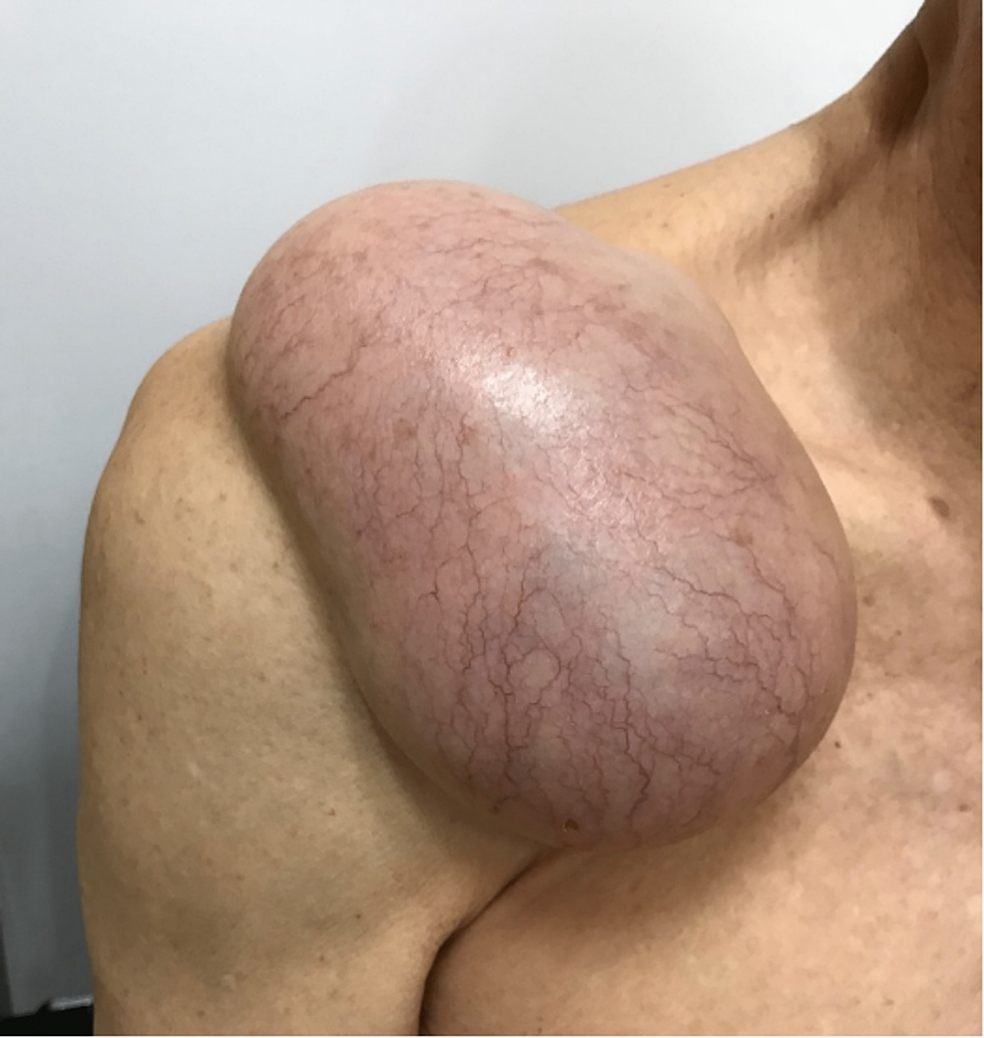
Clinical photograph showing recurrence of right shoulder ACJ cyst with progressive enlargement in size post needle aspiration. ACJ: acromioclavicular joint

The patient was reviewed 2 months later with a recurrence of the ACJ cyst. The cyst was larger compared to the first clinical presentation. Given the recurrence, surgery was recommended to the patient. However, the patient was keen on non-surgical treatment and opted for a repeat needle aspiration procedure. The repeat aspiration yielded 180ml of gelatinous fluid. Recurrence was noted less than a month later. Repeated aspirations were performed for the patient as he continued to decline surgical management for his condition. The repeated aspirations performed at subsequent 4-month interval follow-ups yielded 150mL and 300mL aspirate volumes, respectively. Despite repeated aspirations, there was a progressive enlargement of the ACJ cyst beyond the size of the initial presentation (Figures 11, 12). Surgical options were re-discussed, and the patient decided to proceed with a right shoulder arthroscopic debridement and rotator cuff repair with distal clavicle excision. The patient was planned for an aspiration of the cyst 2 weeks prior to surgery to reduce skin tension. Unfortunately, the patient died from an unrelated condition before the scheduled surgery date.

## Discussion

The ACJ cyst was first described by Craig et al. in 1984 in association with the geyser sign [16]. Hiller et al. further classified ACJ cysts into types I and II based on the postulated pathoanatomy [3]. Type I cysts are isolated to the ACJ and are formed secondary to ACJ degeneration leading to increased fluid production and accumulation in the joint. Type II cysts are associated with rotator cuff tears and subsequent superior migration of the humeral head with disruption of the inferior ACJ capsule, leading to the communication of joint fluid between the glenohumeral joint and ACJ [3]. Our patient presented with a large type II ACJ cyst that was recalcitrant to repeated aspiration and grew larger over the clinical course.

ACJ cysts are rare, and the existing literature is limited to case reports and case series. Ticker et al. reported an incidence of ganglion cysts of the shoulder of 1% [17]. The ideal management of type II ACJ cysts remains controversial. The management options include watchful waiting [12-14], aspiration [1,3,5,9] or corticosteroid injections [6,10,11], and surgical management [2]. A recent systematic review by Christodoulou et al. showed that while surgical management is often utilized, there is a wide variety in the types of surgery performed. He found that there were 68 operative techniques performed on 36 patients, either as single or combined procedures. The 2 most common surgical procedures performed were cyst excision and distal clavicle excision. While surgery appears to lower recurrence rates [2], there is currently insufficient data to determine the ideal surgical treatment. Some authors have proposed that repairing the rotator cuff with cyst excision and acromioplasty could address the ACJ cyst and underlying rotator cuff pathology [1,12,16]. In cases where the rotator cuff is irreparable, a lateral clavicle excision and ACJ resection are preferred to address the pinch valve effect [18]. The former technique showed no recurrence 6 months [19] and 1 year [8] post-operation, while the latter technique showed similar results at the 3-month [4], 6-months [6], 1-year [7], 2-year [15], 3-years [2] post operation mark.

There is a paucity of literature on the effectiveness of conservative management of type II ACJ cysts. A possible option is watchful waiting. There are very few cases of spontaneous resolution, and the time to resolution ranges from 2 months [12] to more than a year [20]. Hartog et al. [12] reported a case of a 3.5cm type II ACJ cyst that underwent spontaneous resolution at the 2-month follow-up after a ‘wait-and-see’ approach. Singh et al. [20] reported a case of a 3cm by 3cm ACJ cyst, which was observed to have enlarged in size at the 9-month follow-up but spontaneously resolved 6 months later. While these cases raise the possibility of spontaneous resolution, the success of watchful waiting appears to be limited.

Needle aspiration and steroid injection are common conservative treatment options. These procedures are simple to perform and provide an immediate reduction in the size of the ACJ cyst. Spinnato et al. performed ultrasound-guided intraarticular steroid injection and observed complete resolution of the ACJ cyst at the 8-month post-procedure follow-up [11]. The main complication of aspiration is recurrence and fistula formation [6,8,15]. Patel et al. [9] reported re-accumulating the ACJ cyst to a lesser extent after needle aspiration. Gumina et al. [10] reported the recurrence of all 4 ACJ cysts treated with aspiration and steroid injection. The author also reported the timeline of recurrence. Within 2 weeks, the author observed rapid recurrence of ACJ cysts in all 4 cases and reaching its original size within a month post aspiration. The author also observed a minimal decrease in pain intensity and reduction of skin tension post aspiration, rendering this procedure ineffective even to treat for symptomatic relief [10]. Several authors [1,15] have postulated that without addressing the underlying rotator cuff pathology to eliminate the pinch valve effect, the ACJ cyst will inevitably recur. Based on current literature, there appears to be no universal cut-off size to favor either aspiration or surgical intervention. The size of ACJ cysts that have failed conservative treatment has ranged from 4cm [15] to 8cm [3].

The current literature focuses mainly on surgical management and postoperative outcomes. Prior interventions, either surgical or non-surgical, are not well-described, and this makes it difficult to reliably-identify predictors of recurrence. In our case, needle aspiration proved to be unsuccessful. Even more critically, there was a gradual enlargement of the cyst over time, resulting in further patient distress and significant implications for future surgical management. Such a large amount of fluid makes arthroplasty challenging for soft tissue management and potential infection.

There are several recommendations that the authors would like to propose based on this case and literature review. Fluid analysis of the aspirate should include cell counts, fluid cultures, and cytopathological studies in larger lesions. In addition, patients who undergo aspiration should be reviewed early to exclude infection, fistula formation, or recurrence. Finally, the authors suggest that if a cyst recurs after initial aspiration, further aspiration should not be attempted, and surgical management should be offered to the patient.

## Conclusions

ACJ cyst is a rare condition frequently associated with massive rotator cuff tears. Post-procedural risk of complications and recurrence is often weighed against the short-term therapeutic effects of needle aspiration in deciding on further conservative management. However, the possibility of continued exacerbation after recurrence, as documented in this study, should raise caution.

This case demonstrates recurrence and continued cyst enlargement after needle aspiration, with implications on patient distress and potentially complicating future attempts at arthroplasty. The authors suggest that if a cyst recurs after initial aspiration, further aspiration should not be attempted, and surgical management should be offered to the patient.

## References

[1] Tshering Vogel DW, Steinbach LS, Hertel R, Bernhard J, Stauffer E, Anderson SE (2005). Acromioclavicular joint cyst: nine cases of a pseudotumor of the shoulder. Skeletal Radiol.

[2] Christodoulou KC, Kakagia DD, Galanis VG, Tsoucalas GI, Fiska AT (2021). Gigantic acromioclavicular joint cyst: presentation and mini review. J Shoulder Elbow Surg.

[3] Hiller AD, Miller JD, Zeller JL (2010). Acromioclavicular joint cyst formation. Clin Anat.

[4] Zhang Y, Old J (2018). Massive acromioclavicular joint cyst with intramuscular extension: case report and review. Case Rep Orthop.

[5] Murena L, D'angelo F, Falvo DA, Vulcano E (2009). Surgical treatment of an aseptic fistulized acromioclavicular joint cyst: a case report and review of the literature. Cases J.

[6] De Maio F, Di Marcantonio A, De Luna V, Caterini A, Tresoldi I, Farsetti P (2020). Synovial cyst of the acromioclavicular joint with and without rotator cuff tear: a case series of two patients. Int J Surg Case Rep.

[7] Purohit S, Keny S, Raja B, Marathe N (2019). Massive acromio-clavicular joint ganglion cyst associated with cuff tear arthropathy and acromioclavicular joint arthritis with normal functional shoulder-a case report. J Clin Orthop Trauma.

[8] Nowak DD, Covey AS, Grant RT, Bigliani LU (2009). Massive acromioclavicular joint cyst. J Shoulder Elbow Surg.

[9] Patel J, Cunha JS (2019). Geyser sign. J Clin Rheumatol.

[10] Gumina S, Candela V, Passaretti D (2016). Acromioclavicular joint cyst in ASA 3-4 patients.whether and how quickly it recurs after aspiration and steroid injection. Acta Orthop Belg.

[11] Spinnato P, Facchini G, Bazzocchi A, Errani C, Marinelli A (2021). Acromioclavicular joint cyst with intramuscular extension presenting as a mass at the base of the neck. J Clin Rheumatol.

[12] de Hartog B, Schimmel JW, Rijk PC (2011). Spontaneous disappearance of an acromioclavicular joint cyst: a case report. Am J Orthop (Belle Mead NJ).

[13] Montet X, Zamorani-Bianchi MP, Mehdizade A, Martinoli C, Bianchi S (2004). Intramuscular ganglion arising from the acromioclavicular joint. Clin Imaging.

[14] McCreesh KM, Riley SJ, Crotty JM (2014). Acromio-clavicular joint cyst associated with a complete rotator cuff tear - a case report. Man Ther.

[15] Tanaka S, Gotoh M, Mitsui Y, Shirachi I, Okawa T, Higuchi F, Shiba N (2017). A case report of an acromioclavicular joint ganglion associated with a rotator cuff tear. Kurume Med J.

[16] Craig EV (1984). The geyser sign and torn rotator cuff: clinical significance and pathomechanics. Clin Orthop Relat Res.

[17] Jonathan BT, Djurasovic M, Strauch RJ, April EW, Pollock RG, Flatow ET, Bigiiani LU (1998). The incidence of ganglion cysts and other variations in anatomy along the course of the suprascapular nerve. J Shoulder Elbow Surg.

[18] Cho CH (2014). Complicated acromioclavicular joint cyst with massive rotator cuff tear. Am J Orthop (Belle Mead NJ).

[19] Marino AJ, Tyrrell PN, El-Houdiri YA, Kelly CP (1998). Acromioclavicular joint cyst and rotator cuff tear. J Shoulder Elbow Surg.

[20] Singh RA, Hay BA, Hay SM (2013). Management of a massive acromioclavicular joint cyst: the geyser sign revisited. Shoulder & Elbow.

